# Combination of Bortezomib and Mitotic Inhibitors Down-Modulate Bcr-Abl and Efficiently Eliminates Tyrosine-Kinase Inhibitor Sensitive and Resistant Bcr-Abl-Positive Leukemic Cells

**DOI:** 10.1371/journal.pone.0077390

**Published:** 2013-10-14

**Authors:** Octavian Bucur, Andreea Lucia Stancu, Ioana Goganau, Stefana Maria Petrescu, Bodvael Pennarun, Thierry Bertomeu, Rajan Dewar, Roya Khosravi-Far

**Affiliations:** 1 Department of Pathology, Harvard Medical School and Beth Israel Deaconess Medical Center, Boston, Massachusetts, United States of America; 2 Institute of Biochemistry of the Romanian Academy, Bucharest, Romania; 3 Carol Davila University of Medicine and Pharmacy, Bucharest, Romania; 4 Biological and Biomedical Sciences Program, Harvard Medical School, Boston, Massachusetts, United States of America;; Medical College of Wisconsin, United States of America

## Abstract

Emergence of resistance to Tyrosine-Kinase Inhibitors (TKIs), such as imatinib, dasatinib and nilotinib, in Chronic Myelogenous Leukemia (CML) demands new therapeutic strategies. We and others have previously established bortezomib, a selective proteasome inhibitor, as an important potential treatment in CML. Here we show that the combined regimens of bortezomib with mitotic inhibitors, such as the microtubule-stabilizing agent Paclitaxel and the PLK1 inhibitor BI2536, efficiently kill TKIs-resistant and -sensitive Bcr-Abl-positive leukemic cells. Combined treatment activates caspases 8, 9 and 3, which correlate with caspase-induced PARP cleavage. These effects are associated with a marked increase in activation of the stress-related MAP kinases p38MAPK and JNK. Interestingly, combined treatment induces a marked decrease in the total and phosphorylated Bcr-Abl protein levels, and inhibits signaling pathways downstream of Bcr-Abl: downregulation of STAT3 and STAT5 phosphorylation and/or total levels and a decrease in phosphorylation of the Bcr-Abl-associated proteins CrkL and Lyn. Moreover, we found that other mitotic inhibitors (Vincristine and Docetaxel), in combination with bortezomib, also suppress the Bcr-Abl-induced pro-survival signals and result in caspase 3 activation. These results open novel possibilities for the treatment of Bcr-Abl-positive leukemias, especially in the imatinib, dasatinib and nilotinib-resistant CML cases.

## Introduction

The t(9:22) chromosomal translocation resulting in the Philadelphia chromosome leads to the expression of the Bcr-Abl fusion protein, which plays a critical role in the pathogenesis and progression of Chronic Myeloid Leukemia (CML), in a subset of Acute Lymphoblastic Leukemia (Ph+ALL; 15% to 30% cases) and occasionally in Acute Myelogenous Leukemia (Ph+AML) [1]. Bcr-Abl functions as a constitutively active kinase, activating critical downstream pathways implicated in proliferation, survival or cell movement, such as: Ras-ERK, PI3K-AKT, JAK-STAT or CrkL/Lyn-dependent pathways [2].

Current inhibitors of Abl kinases, such as imatinib mesylate (imatinib), dasatinib or nilotinib have shown great potential in the treatment of CML. However, the emergence of resistance and residual disease eventually lead to CML progression [3]. Imatinib, dasatinib or nilotinib resistance may emerge through Bcr-Abl mutations (such as T315I) and/or Bcr-Abl amplification [4]. Moroever, while a recently approved TKI, Ponatinib, is effective in patients with T315I mutation, cardiovascular, cerebrovascular and peripheral vascular thrombosis, including fatal myocardial infarction and stroke, have occurred in ponatinib-treated patients [5]. Thus, novel therapeutic strategies that target both imatinib-, dasatinib- or nilotinib-resistant and -sensitive Bcr-Abl-positive leukemias, such as CML, need to be developed.

We and others have previously shown that bortezomib (velcade, PS-341), a selective proteasome inhibitor (approved by European Medicines Agency & US Food and Drug Administration (FDA) for the treatment of multiple myeloma and mantle cell lymphoma) efficiently inhibits survival and induces apoptosis in imatinib-resistant Bcr-Abl cells [1]. Bortezomib significantly reduces the signs of CML-like disease in Bcr-Abl transduced mice [1]. Moreover, we also reported that bortezomib treatment caused remission in a patient with Bcr-Abl positive Acute Lymphoblastic Leukemia (ALL), refractory to standard therapies [6]. An excellent response with a complete remission, maintained for more than 4 years since the patient’s initial diagnosis and beginning of the treatment was observed [6]. Based on these results, more than five different clinical trials have been initiated, using bortezomib alone or in combination with other drugs for the treatment of CML and/or Ph+ALL [7,8]. Thus, bortezomib is a promising treatment in Bcr-Abl-positive leukemias.

An interesting study suggested that bortezomib in combination with the cyclin-dependent kinase (CDK) inhibitor flavopiridol synergizes to induce apoptosis in CML cells [4]. Flavopiridol causes an inhibition of the cell cycle in G1 or G2, based on the inhibition of CDK [4]. Other studies have shown that leukemic cells are particularly sensitive when survival pathway inhibitors are combined with mitotic inhibitors [9]. Moreover, combination of bortezomib with mitotic inhibitors (such as paclitaxel) are currently in clinical trials for the treatment of non-small-cell lung carcinoma (NSCLC) and other solid tumors [10]. Thus, we hypothesized that a strategy based on the combined treatment with bortezomib and mitotic inhibitors for the treatment of Bcr-Abl-positive leukemias may be promising. Especially important might be to determine the effectiveness of this strategy in TKIs-resistant Bcr-Abl-positive cases.

Paclitaxel (Taxol), a mitotic inhibitor drug acting by stabilization of microtubules [11], is FDA approved for the treatment of lung, ovarian, breast cancers and advanced forms of Kaposi’s sarcoma. Paclitaxel is now in clinical trials for the treatment of CML [7,11]. *However, to our knowledge, there are no clinical trials or published studies employing the combined bortezomib and paclitaxel regimen for the treatment of Bcr-Abl-positive CML*. Such a combination, if synergistic in inducing apoptosis in Bcr-Abl-positive cells, would significantly decrease the dose of each compound necessary to achieve a therapeutic effect. 

Here we demonstrate that bortezomib, in combination with the mitotic inhibitor paclitaxel, efficiently kill TKIs-resistant and -sensitive Bcr-Abl-positive leukemic cells. In addition, bortezomib in combination with either paclitaxel or BI 2536, another mitotic inhibitor that inhibits PLK1, induces a marked downregulation of total and phosphorylated Bcr-Abl protein levels, thus downregulating the critical Bcr-Abl downstream signaling pathways and activating caspases. Similarly, bortezomib, in combination with other mitotic inhibitors (vincristine and docetaxel), is able to decrease Bcr-Abl activity and increase caspase activation. Taken together, our findings unravel a novel promising treatment for TKIs-resistant and sensitive CML cases, as well as other Bcr-Abl positive leukemias.

## Materials and Methods

### Cell lines and cell culture reagents

K562 and LAMA84 cell lines were purchased from ATCC. The K562 imatinib-resistant cell lines (K562-R) and LAMA84 imatinib-resistant cell lines (LAMA-R) were generated in our laboratory, by incubation of the K562 and LAMA84 cells, respectively, with increasing concentrations of imatinib mesylate for 3-4 weeks, as described in earlier studies [12]. The K562-R and LAMA84-R cells were then continuously cultured in 1μM imatinib mesylate for the course of the experiments. 

Baf3 cells containing either the control vector pMSCV, pMSCV-Bcr-Abl or Baf3 Bcr-Abl T315I cells have been previously described (see Acknowledgements section for details).

All cell lines were grown in RPMI 1640 medium (Mediatech Inc., VA, USA, Cat. # 10-040-CV) supplemented with 10% fetal bovine serum (FBS, HyClone, Cat. # SH30088.03). 2mM L-glutamine (Mediatech, VA, USA, Cat. # 25-005-CI) and 100 units/ml Penicillin/ 100 μg/ml Streptomycin (Mediatech Inc., VA, USA, Cat. # 30-001-CI). Cells were passed every 2-3 days and used in experiments during their logarithmic growth phase.

### Cellular treatments

Bortezomib (also named Velcade, PS-341, Millennium Pharmaceuticals, Cambridge, MA, USA) and imatinib mesylate (also named Gleevec, STI-571, Novartis, Basel, Switzerland) were purchased from the Beth Israel Deaconess Medical Center Pharmacy (approved for research purposes only). The sensitivity of K562, K562-R, LAMA84, LAMA84-R, Baf3 Bcr-Abl, and Baf3 Bcr-Abl T315I cell lines to the most used Bcr-Abl inhibitors in CML: imatinib, dasatinib (Santa Cruz Biotechnology, CA, USA, Cat. # sc-218081) and nilotinib (Santa Cruz Biotechnology, CA, USA, Cat. # sc-202245) was investigated by treating these cells with increasing concentrations of each drug and measuring cell death using an automated Trypan Blue exclusion method (see Figures S2, S3 and S4).

**Figure 1:**
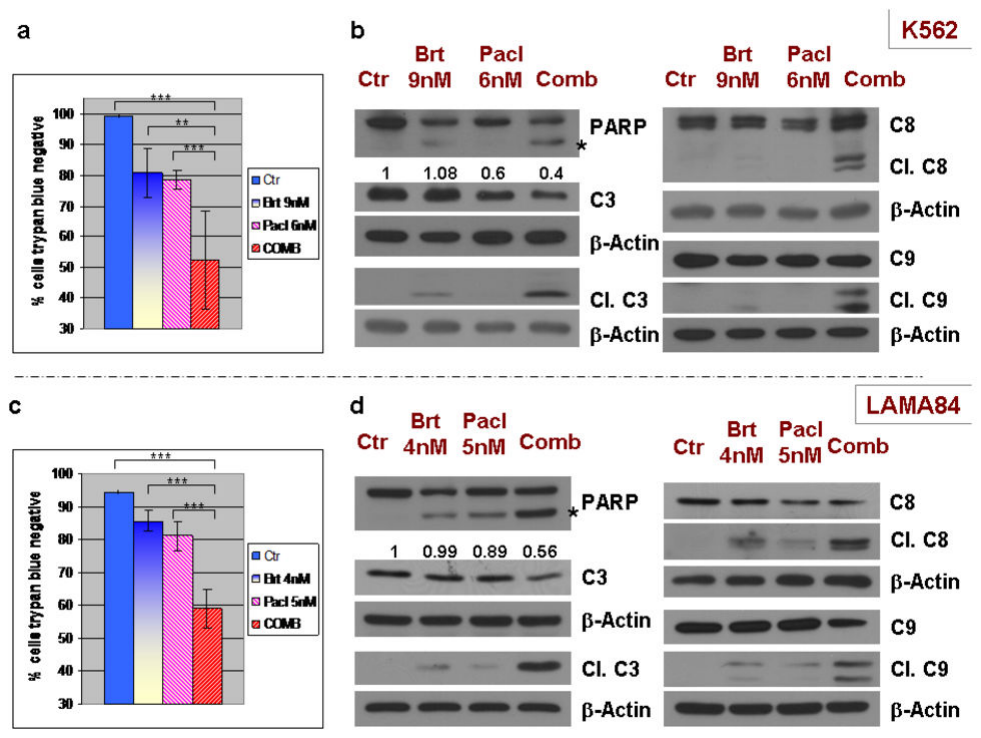
Combined treatment of bortezomib and paclitaxel efficiently activates caspases and induces cell death in human leukemic Bcr-Abl-positive K562 and LAMA84 cell lines. **A**. K562 leukemic cells were exposed to bortezomib (9nM) with or without paclitaxel (6nM) for 48h. The percentage of cell death was measured with an automated Trypan Blue exclusion method. The combination significantly increased the number of Trypan Blue-positive cells, compared with each drug used alone. The results represent the mean +/- standard deviations (SDs) of 4 measurements/condition for the representative Western blot experiment presented in (B). **B**. K562 leukemic cells were treated with 9nM bortezomib and 6nM paclitaxel for 48h, followed by detection of the cleaved fragments of caspase 3, and of PARP cleavage. The combined regimen induced significant cleavage of caspases 3, 8 & 9, and PARP, implying caspase activation. **C**. LAMA84 leukemic cells were exposed to bortezomib (4nM) with or without paclitaxel (5nM), for 48h. The percentage of cell death was measured with an automated Trypan Blue exclusion method. The combination significantly increased the number of Trypan Blue-positive cells, compared with each drug used alone. The results represent the mean +/- standard deviations (SDs) of 5 measurements/condition for the representative Western blot experiment presented in (D). **D**. LAMA84 leukemic cells were treated with 4nM bortezomib and 5nM paclitaxel for 48h, followed by detection of the cleaved fragments of caspase 3, caspase 8, caspase 9 and of PARP cleavage. The combined regimen significantly enhanced the cleavage of caspases 3, 8, 9 and PARP, suggesting caspase activation. At least 3 separate experiments were performed in each case. β-Actin was used as a loading control; “***” = p<0.0001; “**” = p<0.01;.

**Figure 2:**
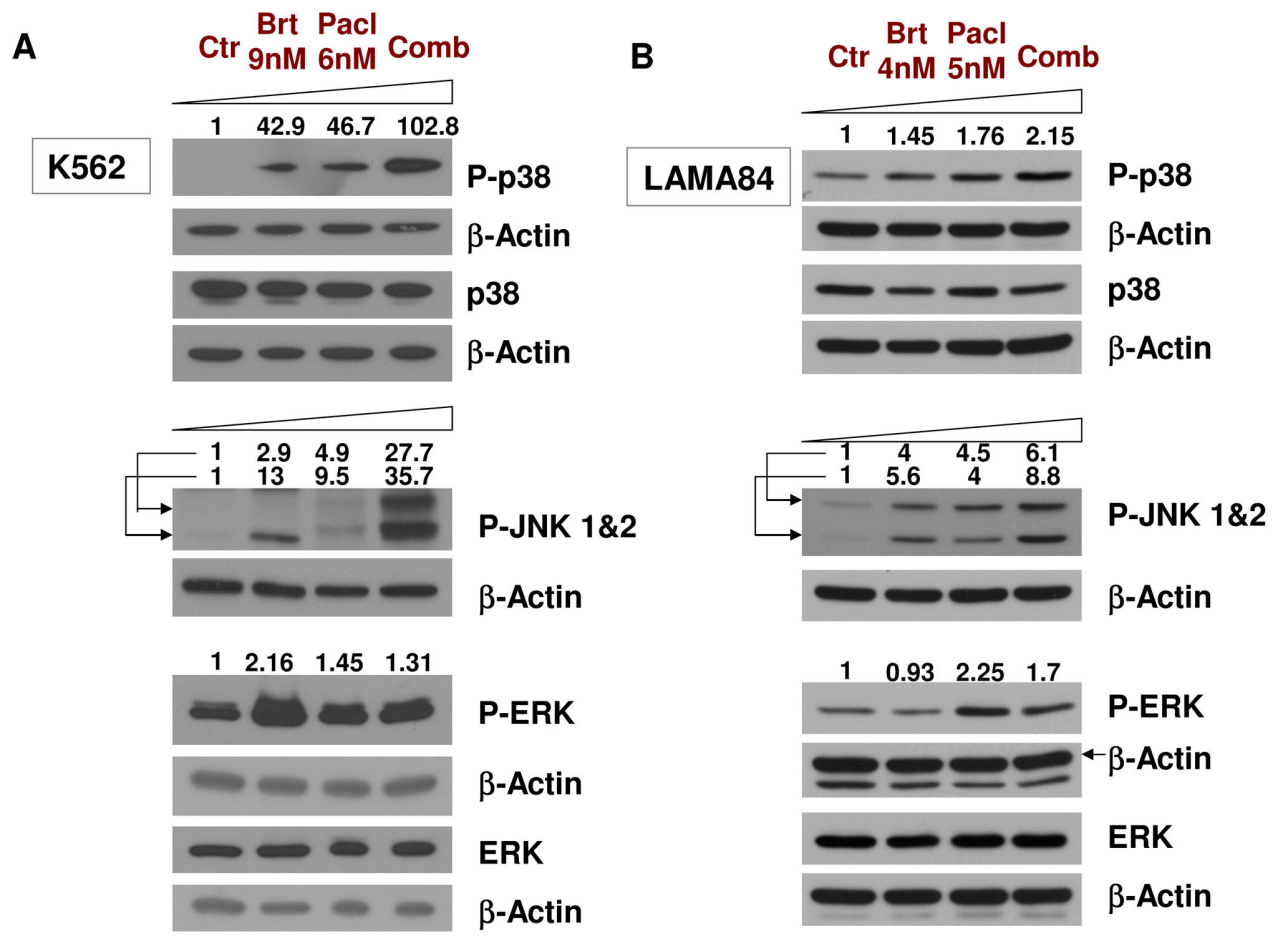
Cotreatment with bortezomib and paclitaxel induces activation of stress-dependent kinases JNK and p38, but not of the cytoprotective ERK kinases in K562 and LAMA84 cell lines. **A**. K562 leukemic cells were treated with 9nM bortezomib and 6nM paclitaxel for 48h, followed by detection of the total and phosphorylated protein levels of p38MAPK, JNK 1&2 and ERK 1&2. The combined regimen induced a strong increase in phosphorylation of the p38MAPK and JNK 1&2, and a slight increase in P-ERK 1&2. **B**. LAMA84 leukemic cells were treated with 4nM bortezomib and 5nM paclitaxel for 48h, followed by detection of the total and phosphorylated protein levels of p38MAPK, JNK 1&2 and ERK 1&2. β-Actin was used as a loading control. One representative experiment from three separate experiments is shown.

**Figure 3:**
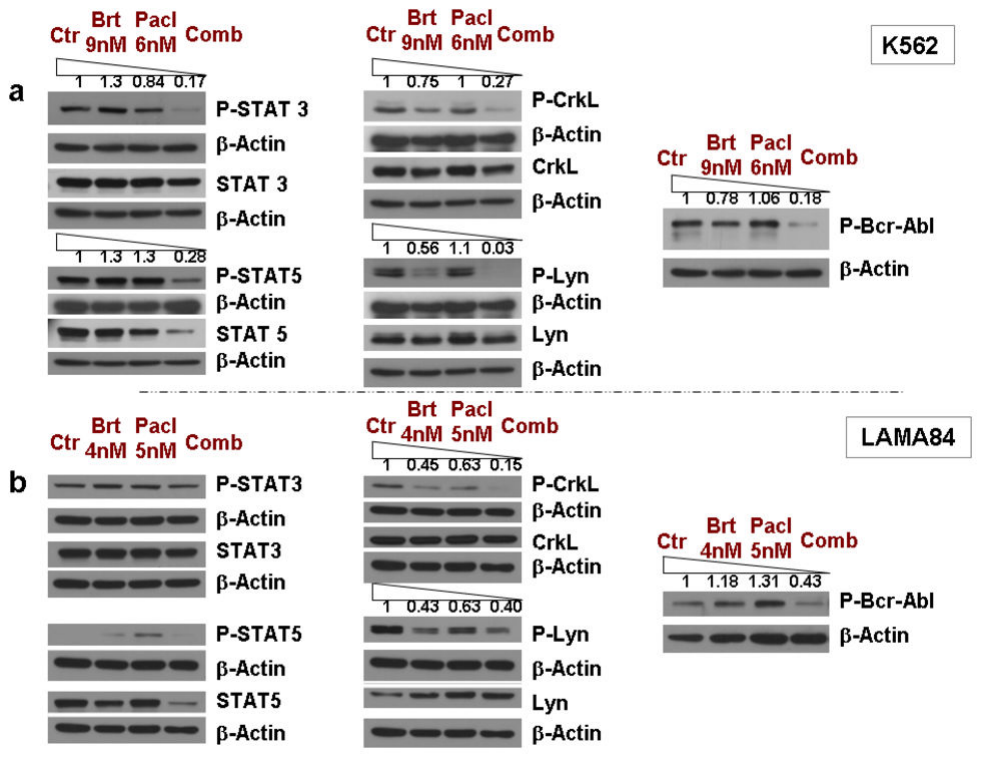
Bortezomib and paclitaxel combined treatment significantly downregulates phosphorylated Bcr-Abl levels, inhibiting the phosphorylation/activity of downstream STAT3/STAT5, CrkL and Lyn kinase-dependent pathways, in K562 & LAMA84 cell lines. **A**. K562 leukemic cells were treated with 9nM bortezomib and 6nM paclitaxel for 48h, followed by detection of the phosphorylated levels of Bcr-Abl, and of the total and phosphorylated protein levels of STAT3, STAT5, CrkL and Lyn. The combined regimen significantly downregulates the phosphorylation of Bcr-Abl, CrkL and Lyn kinases, the total levels and phosphorylation of STAT5 and the phosphorylation of STAT3 transcription factors. **B**. LAMA84 leukemic cells were treated with 4nM bortezomib and 5nM paclitaxel for 48h, followed by detection of the phosphorylated levels of Bcr-Abl, and of the total and phosphorylated levels of STAT3, STAT5, CrkL and Lyn. The combined regimen significantly downregulates the phosphorylation of Bcr-Abl, CrkL and Lyn kinases, and the total levels and phosphorylation of STAT5. β-Actin was used as a loading control. One representative experiment from three separate experiments is shown.

**Figure 4:**
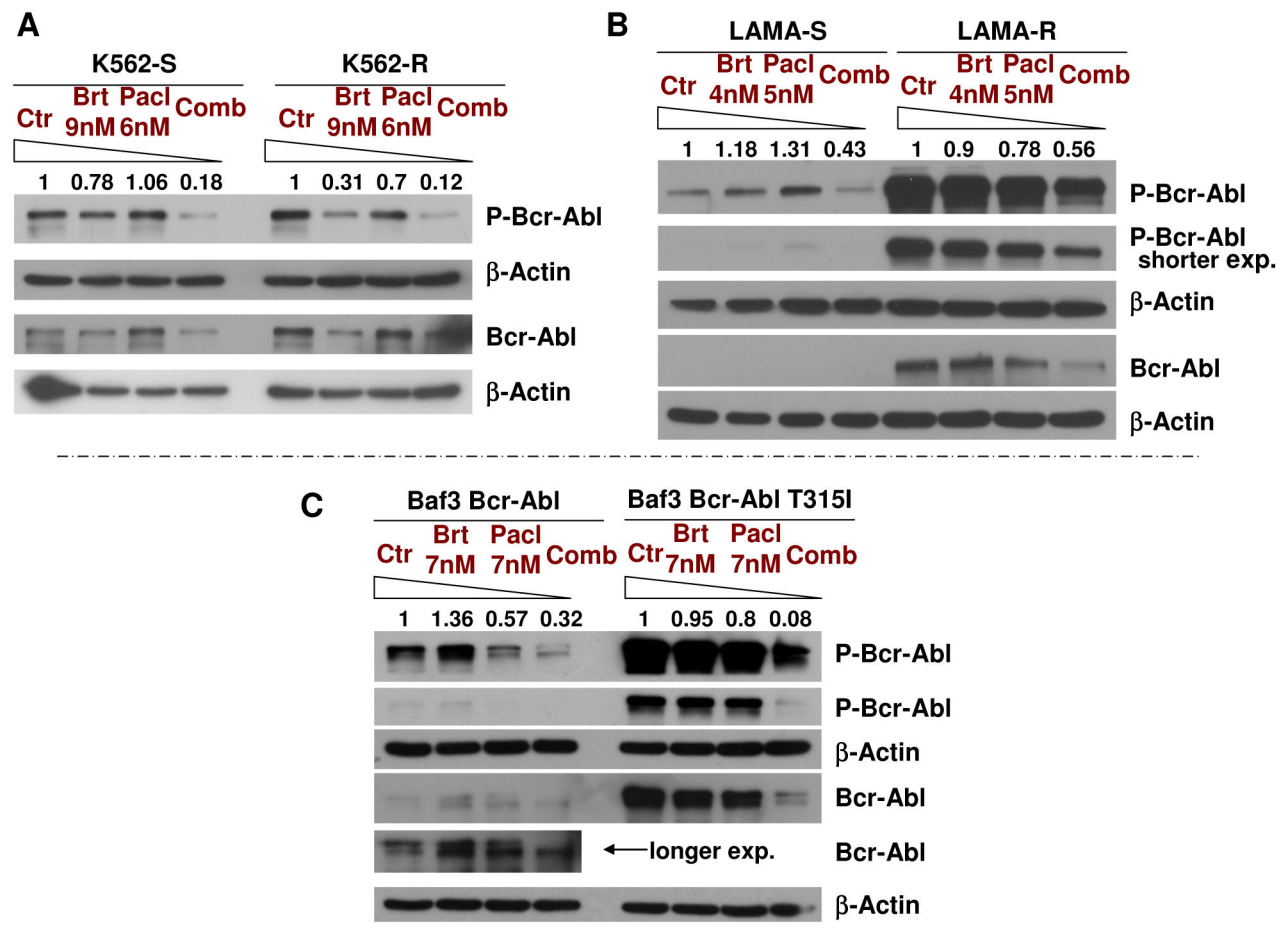
Bortezomib/paclitaxel combination induces downregulation of the total levels and phosphorylation of Bcr-Abl in TKIs-resistant K562-R, LAMA84-R and Baf3 Bcr-Abl T315I, displaying increased Bcr-Abl expression/activity and/or T315I mutation. **A**. K562 (K562-S) and K562-R leukemic cells were treated with 9nM bortezomib and 6nM paclitaxel for 48h, followed by detection of the total levels and phosphorylation of Bcr-Abl. The combined regimen significantly decreases the phosphorylation of Bcr-Abl in both cell lines. **B**. LAMA84 (LAMA84-S) and LAMA84-R leukemic cells were treated with 4nM bortezomib and 5nM paclitaxel for 48h, followed by detection of the total levels and phosphorylation of Bcr-Abl. The combined regimen downregulates the total levels and phosphorylation of Bcr-Abl in LAMA84-R and phosphorylation of Bcr-Abl in LAMA84-S. **C**. Baf3 Bcr-Abl and Baf3 Bcr-Abl T315I cell lines were treated with 7nM bortezomib and 7nM paclitaxel for 48h, followed by detection of the total levels and phosphorylation of Bcr-Abl. The combined regimen decreases the phosphorylation of Bcr-Abl in both cell lines. β-Actin was used as a loading control. One representative experiment from several separate experiments is shown.

**Figure 5:**
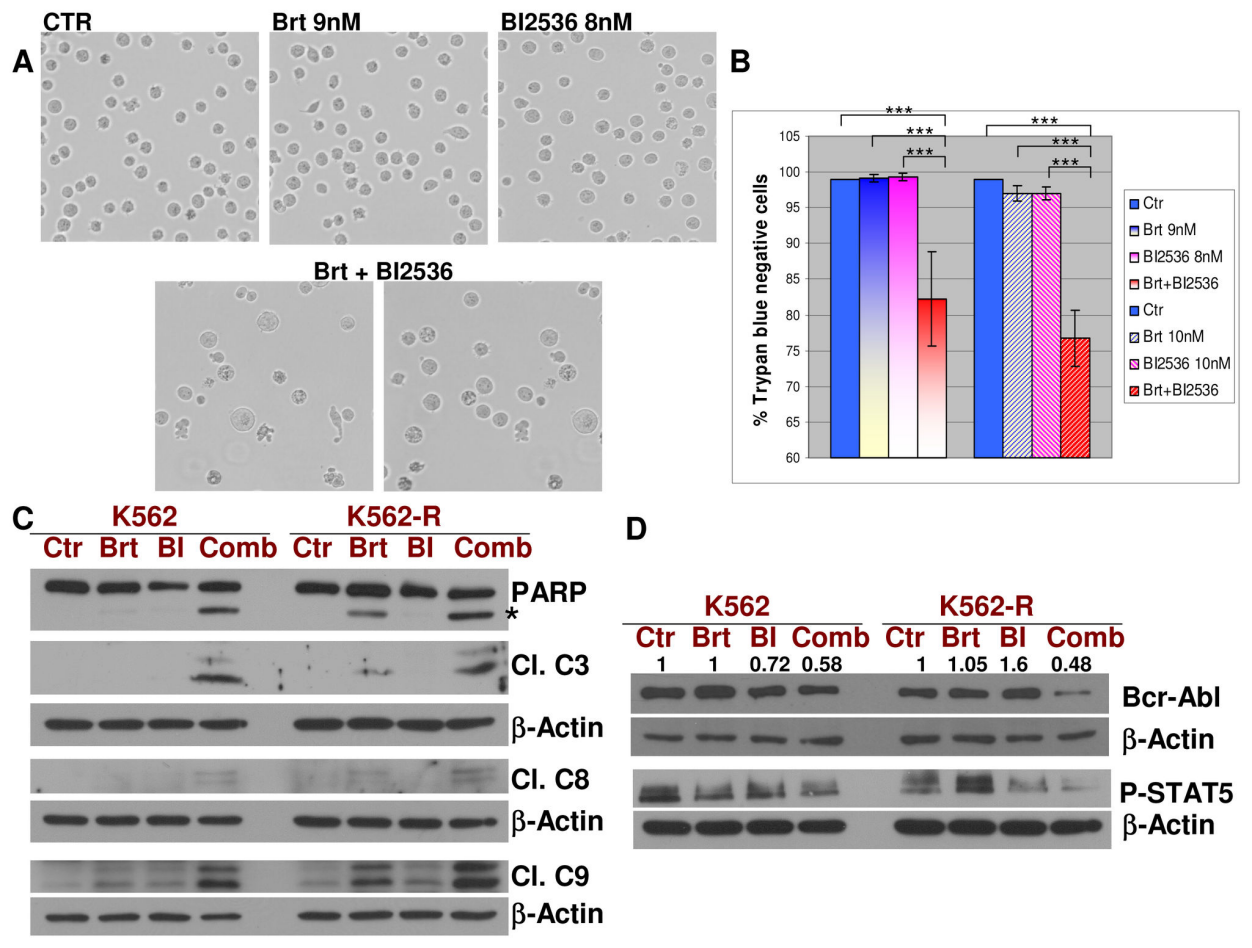
Bortezomib in combination with the PLK1 inhibitor BI 2536 induces a significant downregulation of the total levels and phosphorylation of Bcr-Abl, a decrease of downstream phosphorylated STAT5 and a caspase-dependent cell death in TKIs-resistant and –sensitive K562 cell lines. **A**. K562 leukemic cells were plated in 75cm^2^ flasks (2 x 10^6^ cells/30 ml/flask) and treated with 9nM bortezomib, 8nM BI 2536 or the combination, for 60h. Phase contrast microscope images of the untreated or treated cells are presented. A Nikon Eclipse TI-S Inverted Microscope was used (10X objective lens). While single treatments did not significantly change the morphology and number of the cells, the combined treatment induced a change in morphology/shape and a decrease in the number of the cells. **B**. K562 leukemic cells were plated in 75cm^2^ flasks (2 x 10^6^ cells/30 ml/flask) and treated with 9nM, 10nM bortezomib, 8nM, 10nM BI 2536 or the combination, for 60h. Viability was measured by Trypan Blue dye exclusion method, using a TC10 Automated Cell Counter (Biorad, USA). The results represent the mean +/- standard deviations (SDs) of 6 measurements/condition for the representative Western blot experiment presented in (C) & (D). **C**. K562 and K562-R cells were treated with 9nM bortezomib and 8nM BI 2536 for 60h, followed by detection of the cleaved caspase 3, caspase 8, caspase 9 and PARP. The combined regimen significantly enhanced this cleavage, suggesting caspase activation in both K562 and K562-R cell lines. **D**. K562 and K562-R cells were treated with 9nM bortezomib and 8nM BI 2536 for 60h, followed by the detection of Bcr-Abl and phosphorylated STAT5. The combined treatment resulted in a marked decrease of the total levels of Bcr-Abl, which correlates with a decrease in the phosphorylation of the downstream STAT5 protein, in both K562 and K562-R cells. β-Actin was used as an internal loading control. A total of three independent experiments were performed. “***” = p<0.0001.

**Figure 6:**
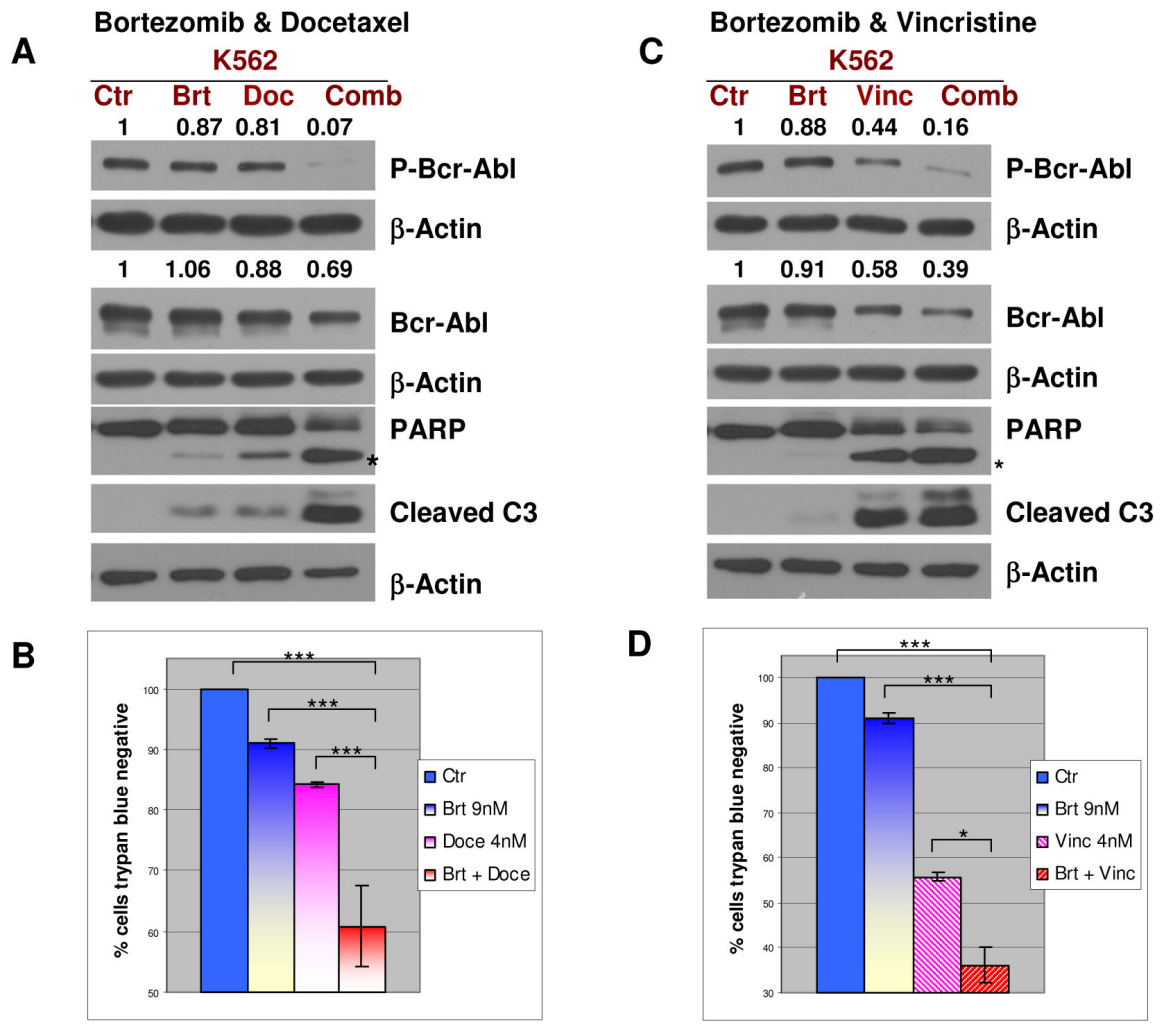
Bortezomib in combination with mitotic inhibitors as a new strategy for CML: combination of bortezomib with other mitotic inhibitors induces downregulation of the Bcr-Abl phosphorylation/total levels and caspase activation in the K562 cell line. **A**. K562 leukemic cells were treated with 9nM bortezomib and 4nM docetaxel for 48h, followed by the detection of total Bcr-Abl levels, phosphorylated Bcr-Abl, cleaved caspase 3 and PARP. The combined treatment resulted in a marked decrease of the total levels and phosphorylation of Bcr-Abl. This effect is associated with an increase in cleavage of both caspase 3 and PARP. **B**. K562 leukemic cells were treated with 9nM bortezomib and 4nM vincristine for 48h, followed by the detection of total Bcr-Abl levels, phosphorylated Bcr-Abl, cleaved caspase 3 and PARP. The combined treatment resulted in a significant decrease in the total levels and phosphorylation of Bcr-Abl compared with individual treatments. This effect is correlated with an increase in cleavage of both caspase 3 and PARP. β-Actin was used as an internal loading control; “***” = p<0.0001; “*” = p<0.05;.

Paclitaxel (Sigma, MO, USA, Cat # T7402), BI 2536 (Selleck Chemicals, TX, USA, Cat. # S1109), docetaxel (Santa Cruz Biotechnology, CA, USA, Cat. # sc-201436) and vincristine (Santa Cruz Biotechnology, CA, USA, Cat. # sc-338734) were used alone or in combination with bortezomib to evaluate the effect of bortezomib in combination with diverse mitotic inhibitors on Bcr-Abl-positive cells (see Figures 1-6 and Figure S1).

### Viability/cell death analysis (automated trypan blue exclusion assay)

Cell death and viability were measured using an automated Trypan Blue exclusion assay. The BioRad TC10 automated cell counter is designed to accurately score Trypan Blue-positive and -negative suspended cells and can determine viability by an automated image analysis algorithm. The TC10 counter demonstrates high reproducibility when cell concentration is within 5x10^4^ - 10^7^ cells/ml (we used and recommend a concentration higher than 10^5^ cells/ml) and the cells measure 6-50 μm in diameter. Briefly, after treating the cells with indicated drugs for the specified time, a volume of 30-40 μl of cells is mixed with an equal volume of Trypan Blue solution (0.4%). 10 μl of this mix is then loaded on a special counting chamber and measured with the BioRad TC10 cell counter after 10 seconds. At least six readings were made for each condition, in each individual experiment. This method was used for the dose-effect experiments and for demonstrating the synergistic effect of the bortezomib and paclitaxel combination.

### Cell death/apoptosis analysis (PE-Annexin V/7-AAD staining)

To confirm the results obtained with Trypan blue assay, K562 cells treated with bortezomib, paclitaxel or the combination were also evaluated by PE-Annexin V/7-AAD staining (PE Annexin V Apoptosis Detection Kit I, BD PharMingen, San Diego, CA) and flow cytometry. 

### Viability analysis (MTT assay)

Viability/proliferation was measured by using the MTT assay (MTT kit, Invitrogen), which is a standard colorimetric assay for measuring the activity of enzymes that reduce MTT to formazan, giving a purple color. Human leukemic K562 cell lines were treated with bortezomib, paclitaxel and combination, using the specified doses, in 96 well plates, at the density of 10,000 cells/well (5 wells were used for each condition) in each individual experiment. After 24 or 48h, MTT assay was performed. The absorbance was measured at 570 nm. 

### Proliferation analysis (BrdU incorporation assay)

Proliferation was measured by using the BD Pharmingen BrdU Flow Cytometry Kit. Briefly, human leukemic cells K562 were treated with bortezomib, paclitaxel or combination, for 40h. Cells were exposed to BrdU for 90 min before fixation and permeabilization. Cells were analyzed by flow cytometry for BrdU incorporation by using an anti-BrdU FITC coupled antibody, following the manufacturer’s instructions.

### Determination of the synergistic effect

The synergistic effect of the combined treatment was established as previously described [13], by using the Chou and Talalay method [14]. This is a generalized method for analyzing the effects of multiple drugs and for determining summation, synergism and/or antagonism. K562, LAMA84 & K562-R cells were treated with increasing concentrations from each drug alone and in combination, maintaining the same concentration ratio of bortezomib/paclitaxel (1/1.5). The value of the Combination Index (CI) using Chou-Talalay method that indicates synergism is CI < 1, while CI = 1 shows an additive effect and CI > 1, antagonism [14].

### Western blot

Suspension cells were washed with PBS and lysed in RIPA buffer. SDS-PAGE and the immunoblotting were performed as previously described [15]. Most of the antibodies employed for probing the Immobilon PVDF membranes were purchased from Cell Signaling Technology (MA, USA) and used at dilutions of 1:1,000 or 1:1,500: c-Abl (# 2862), P-Bcr Y177 (# 3901), CrkL 32H4 (# 3182), P-CrkL Y207 (# 3181), Lyn (# 2732), P-Lyn Y507 (# 2731), Stat 5 (# 9363), P-Stat 5 Y694 (# 9359), Stat 3 (# 9132), P-Stat 3 S727 (# 9134), p38 MAPK (# 9212), P-p38 MAPK T180/Y182 (# 4511), SAPK/JNK 56G8 (# 9258), P-SAPK/JNK T183/Y185 (# 9251), p44/42 MAPK (ERK 1/2) (# 4695), P-p44/42 MAPK T202/Y204 (# 4370), PARP (# 9542), Caspase 3 (# 9662), Cleaved Caspase3 (# 9664), Caspase 8 1C12 (# 9746), Cleaved Caspase 8 (# 9496), Caspase 9 (# 9502). Anti-β-Actin antibody (clone AC-15) (# A5441) was purchased from Sigma (MO, USA) and used at a dilution of 1:20,000.

 The quantitative analysis of the protein expression levels (normalized to beta-Actin) was done using ImageJ software, following the developer’s instructions (the background was substracted and the image was inverted, to minimalize the errors). 

### Photomicrograph and image processing

Phase contrast/bright field microscope images were taken using a Nikon Eclipse TI-S Inverted Microscope (4x or 10x objective lenses) and by using QCapture 2.99.5 software (2009, Quantitative Imaging Corporation).

### Statistical analysis

The values presented in the graphs of this manuscript represent the means +/- SD. Student *t* test (2 tailed distribution, unpaired) was employed to analyze the significance of the differences between experimental variables (“*” = p < 0.05; “**” = p<0.01; “***” = p<0.0001). See Figure legends for details of each experiment. 

## Results

### Combined treatment with bortezomib and paclitaxel efficiently activates caspases and induces cell death in human leukemic Bcr-Abl-positive K562 and LAMA84 cell lines

Dose-effect curves for single treatments with bortezomib [1,4] and paclitaxel [16] were previously determined in K562, LAMA84 or Baf3 Bcr-Abl cells by us or other groups. To assess bortezomib/paclitaxel interaction in Bcr-Abl positive cells, K562 and LAMA84 were exposed to 9nM (K562) or 4nM (LAMA84) bortezomib, alone or in combination with 6nM (K562) or 5nM (LAMA84) paclitaxel, for 48h. Specifically, 9nM bortezomib in combination with 6nM paclitaxel induces 48% cell death in K562, while each treatment alone induces less than 21% cell death, as measured with Trypan Blue exclusion assay (Figure 1a). The combined treatment results in a decrease in pro-caspase 3, as well as a significant increase in cleavage of the initiator caspases 8 and 9 and the effector caspase 3, in K562 cells (Figure 1b). This indicates activation of caspases, and suggests the involvement of both the extrinsic and intrinsic pathways of apoptosis. In order to analyze the direct activity of caspases on their known substrates, we have detected the Poly(ADP-Ribose) Polymerase (PARP) cleavage by Western blot. PARP is a well established substrate for many caspases [17]. PARP cleavage was significantly enhanced in the combined treatment compared with each treatment alone underscoring caspase activation during bortezomib/paclitaxel-induced cell death (Figure 1b, cleaved fragment marked with“*”). Similarly, LAMA84 cell treated with 4nM bortezomib in combination with 5nM paclitaxel resulted in 41% cell death, while single treatments induce 15% (bortezomib) and 19% (paclitaxel) cell death (Figure 1c). Combined treatment induced a decrease of pro-caspase 3 and a significant increase in cleaved fragments of caspases 3, 8, 9. These results correlate with an increase in PARP cleavage (Figure 1d, cleaved fragment marked with “*”)

 We further confirmed these results by analyzing bortezomib/paclitaxel-induced cell death/apoptosis with Annexin V/7AAD and the effect on viability/proliferation with MTT assay, in K562 (Figure S1a and b). Moreover, the effect on proliferation was also determined. Notably, while in the control and bortezomib treatments, 90min of BrdU exposure resulted in a BrdU incorporation of 65.1% and 60.1% cells respectively, in paclitaxel treated cells and combination the proliferation is significantly decreased (19.7% and 29.2% respectively). Combined treatment has less effect on proliferation compared to paclitaxel alone, suggesting that combined regimen is acting mainly by inducing cell death, and only partially by inhibiting proliferation (Figure S1c).

 In addition, dose-effect analysis of this caspase-induced cell death by Trypan Blue exclusion in K562 and LAMA84 cells revealed a combination index (CI) with values lower than 1, which shows that bortezomib and paclitaxel act synergistically in inducing cell death (Figure S2a and b).

### Co-treatment with bortezomib and paclitaxel induces activation of stress-dependent kinases JNK and p38 in K562 and LAMA84 cell lines

Previous reports have shown that Bcr-Abl and various treatments (such as imatinib, dasatinib, nilotinib and bortezomib) are able to modulate the MAPK cascades [3,4]. In order to evaluate the bortezomib and paclitaxel interactions with MAPK signaling pathways, we analyzed the effect of bortezomib and paclitaxel combined treatment on activation p38MAPK, JNK and ERK. While bortezomib or paclitaxel alone are able to induce a slight increase in phosphorylated p38MAPK, combined treatment leads to a robust p38MAPK activation, with more than 100x increase in K562 and 2x increase in LAMA84, compared with the control. Similar to other reports [4], bortezomib alone is able to induce an increase in JNK phosphorylation (activation) (Figure 2a & b). JNK activation is further increased when bortezomib is used in combination with paclitaxel, in both K562 and LAMA84 cell lines (Figure 2a and b). Treatment with bortezomib, paclitaxel or their combination did also increase the phosphorylation of ERK1/2. However, the increase of P-ERK1/2 is modest: 1.31x in K562 and 1.7x in LAMA84. Notably, when slightly lower concentrations of bortezomib (7.5-8nM) and paclitaxel (5nM) are used in K562, the combined treatment does not result in an increase in P-ERK1/2 (Figure S3a), while levels of P-p38 (Figure S3b) and cleaved caspase 3 (data not shown) are significantly increased. These findings suggest that the bortezomib/paclitaxel regimen induces a significant activation of stress-dependent kinases (JNK & p38 MAPK), while the cytoprotective kinases ERK1 & ERK2 are only activated at higher concentrations (Figure 2). 

### Bortezomib and Paclitaxel combined treatment significantly downregulates phosphorylated Bcr-Abl levels, inhibiting the phosphorylation/activity of downstream STAT3/STAT5, CrkL and Lyn-dependent pathways (K562 & LAMA84 cell lines)

The combined treatment with bortezomib and paclitaxel decreases the phosphorylation (activity) of Bcr-Abl in both K562 and LAMA84 cell lines (Figure 3, right panels). This intriguing result suggests that the combination targets Bcr-Abl through a novel mechanism. 

Previous reports suggest that Bcr-Abl is able to induce activation of multiple downstream pathways through phosphorylation of the CrkL adaptor protein, phosphorylation of the Lyn kinase, and activation of the JAK/STAT pathway by phosphorylation of STAT proteins [18-20]. Thus, we evaluated the phosphorylation of these downstream factors. The combined treatment with bortezomib and paclitaxel clearly decreases the phosphorylation of CrkL and Lyn, in both K562 and LAMA84 leukemic cell lines (Figure 3). These proteins are known to be activated by phosphorylation and mediate the pathways implicated in cell transformation and movement (CrkL) [18] or cell survival (Lyn) [19]. Additionally, bortezomib and paclitaxel combination induces the downregulation of total levels and phosphorylation of STAT5 (K562 & LAMA84 cells) and of phosphorylation of STAT3 (K562) (Figure 3). STAT transcription factors act downstream of Bcr-Abl in mediating proliferation/survival, transformation and anti-apoptotic signals, through a number of already established apoptotic/cell cycle related proteins, such as: Bcl-2, Mcl-1, Cyclins or c-Myc [20,21].

Taken together, we demonstrate that the bortezomib/paclitaxel combination effectively inhibits the activity of Bcr-Abl and of its downstream signaling mediators. 

### Bortezomib/paclitaxel combination induces a downregulation of the phosphorylation of Bcr-Abl in TKIs-sensitive (K562, LAMA84, Baf3 Bcr-Abl) and TKIs-resistant (K562-R, LAMA84-R, Baf3 Bcr-Abl T315I) cell lines

In order to evaluate if the combined bortezomib/paclitaxel regimen can efficiently shut down Bcr-Abl and induce cell death in Bcr-Abl-positive leukemic cell lines that are resistant to imatinib, we developed two different cell lines derived from K562 (K562-R) and LAMA84 (LAMA84-R) cell lines, which are resistant to 1μM imatinib. Additionally, we have used the Baf3 Bcr-Abl T315I cell line, a Baf3 derivative resistant to 1μM imatinib. Importantly, these three cell lines also demonstrate increased resistance to dasatinib and nilotinib (Figures S4, 5 and 6).

 The effects of 9nM bortezomib, 6nM paclitaxel or both drugs in combination were evaluated in both parental K562 (imatinib-sensitive) and TKIs-resistant K562-R cells after 48h of treatment. Similar to K562, K562-R cells are synergistically killed by bortezomib/paclitaxel regimen, as shown by a CI lower than 1 (when affected fraction fa> 0.18) (Figure S7). Notably, combined treatment with bortezomib and paclitaxel strongly decreases phosphorylation of Bcr-Abl, in both K562 (K562-S) and K562-R cell lines (Figure 4a). 

 The effects of 4nM bortezomib, 5nM paclitaxel or both drugs in combination for 48h were also evaluated in both parental LAMA84 (imatinib-sensitive) and TKIs-resistant LAMA84-R cells. Interestingly, the LAMA84-R cells show a significant increase in total levels and phosphorylation of Bcr-Abl oncoprotein when compared with parental LAMA84 cells (LAMA84-S). This suggests that these cells adapted to resist imatinib, dasatinib and nilotinib treatments through the upregulation of Bcr-Abl levels and activity. Combined treatment with bortezomib and paclitaxel was able to downregulate total levels and phosphorylation of Bcr-Abl in LAMA84-R cell lines (Figure 4b). 

 The T315I point mutation in Bcr-Abl is known to confer resistance to imatinib, dasatinib, nilotinib, and bosutinib [22]. It is therefore important to test whether the combined treatment of bortezomib and paclitaxel is also active on cells expressing Bcr-Abl with the T315I mutation. While 7nM bortezomib and 7nM paclitaxel alone did not significantly affect the total levels and phosphorylation of Bcr-Abl T315I in the Baf3 Bcr-Abl T315I cells, the combined treatment was highly effective in decreasing the total levels and phosphorylation of Bcr-Abl T315I (Figure 4c). Our results show that bortezomib and paclitaxel combined treatment is able to target the TKIs-resistant cell lines with the T315I mutation in Bcr-Abl.

### Bortezomib in combination with the PLK1 inhibitor BI 2536 induces a significant downregulation of the total levels and phosphorylation of Bcr-Abl, a decrease of downstream phosphorylated STAT5 and caspase-dependent cell death in imatinib-, dasatinib- and nilotinib-resistant and -sensitive K562 cell lines

To determine if bortezomib in combination with other known mitotic inhibitors can result in similar inhibition of Bcr-Abl activity and downstream signaling, we analyzed the combined effect of bortezomib with the known PLK1 inhibitor BI 2536. PLK1 is a well conserved kinase, critical in all phases of the mitosis [23]. A previous report suggested that BI 2536 has a growth inhibitory effect on Bcr-Abl-positive cells, which is not amplified by bortezomib after 16h of co-treatment [23]. Here we show that while each treatment alone at 9nM or 10nM bortezomib and 8nM or 10nM BI 2536 does not significantly induce cell death in K562 as measured by the Trypan Blue exclusion method, the combined treatment resulted in a significant decrease in cell number and in the percentage of the viable cells (Figure 5a and b). In addition, the prolonged (60h) co-treatment with 9nM bortezomib and 8nM BI 2536 is effective in cleaving initiator caspases 8, 9, effector caspase 3 and caspase-substrate PARP, in both K562 and K562-R cells (Figure 5c). Moreover, the combined treatment also resulted in an efficient decrease of the total levels of Bcr-Abl, which correlates with a decrease in the phosphorylation of the downstream STAT5 protein, in both K562 and K562-R cells (Figure 5d).

### Bortezomib in combination with mitotic inhibitors as a novel general strategy in CML: combination of bortezomib with other mitotic inhibitors induces downregulation of the Bcr-Abl phosphorylation/total levels and caspase activation in K562

The combined effects of bortezomib with the mitotic inhibitors docetaxel and vincristine were also tested. Similar to paclitaxel, docetaxel induces mitotic arrest by stabilizing microtubules [24]. The effect of 9nM bortezomib alone, 4nM docetaxel alone, or both drugs in combination for 48h, was evaluated in K562. The combined treatment resulted in a significant decrease of total levels and phosphorylation of Bcr-Abl, and in an increase in caspase 3 cleavage (Figure 6a). 

By contrast, vincristine induces mitotic arrest by destabilizing microtubules [25]. 9nM bortezomib and 4nM vincristine combination induces a decrease in total levels and phosphorylated Bcr-Abl, as well as an increase in caspase 3 cleavage, the effects being higher than in singular treatments (Figure 6b). Moreover, the combinations of bortezomib with docetaxel or vincristine resulted in a significant and higher increase in cell death compared with individual treatments (Figure 6c & 6d).

## Discussion

**Figure 7:**
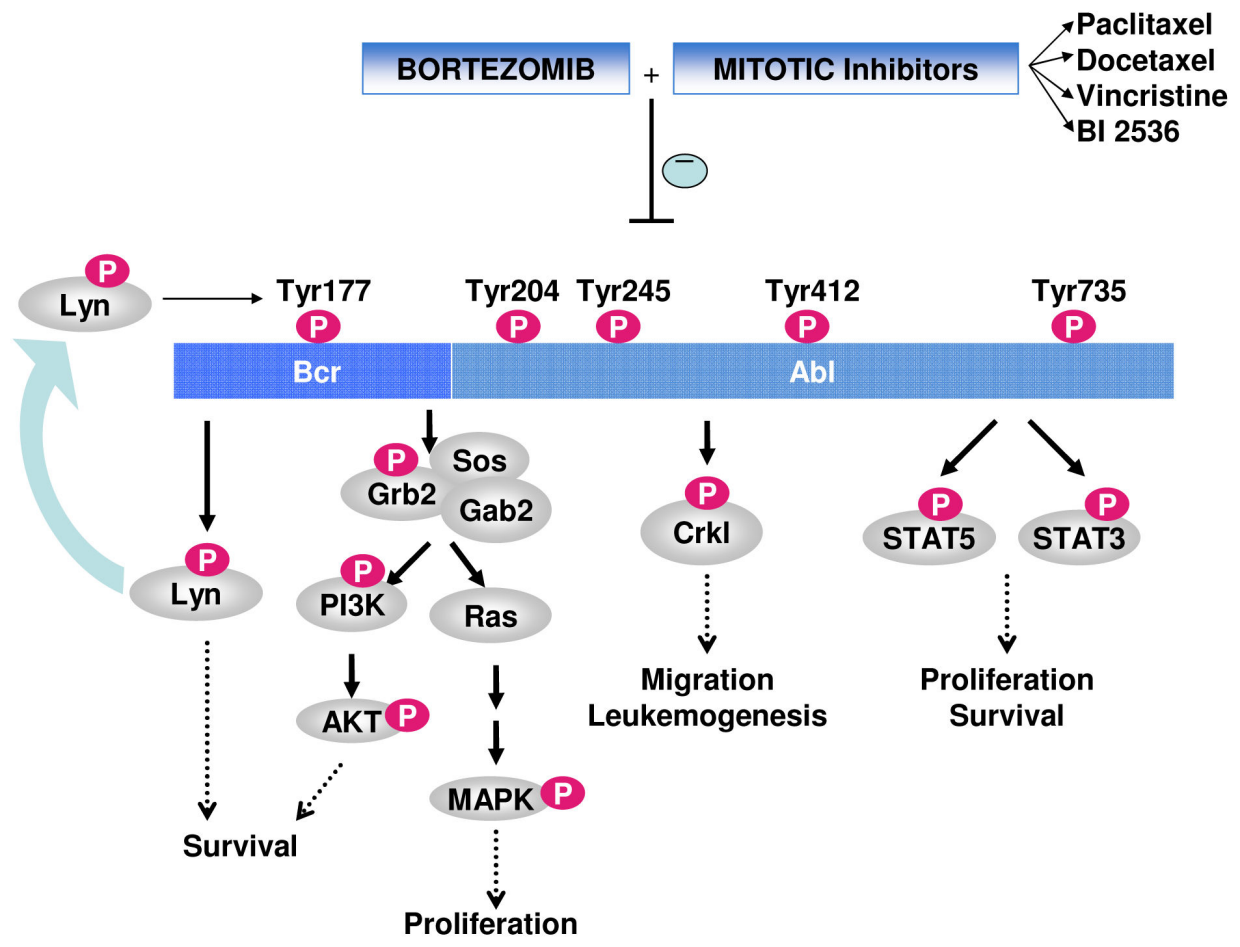
Bortezomib in combination with mitotic inhibitors as a novel strategy for CML: a model of the combined treatments-induced cell death. Our results show that bortezomib in combination with several mitotic inhibitors, known to suppress mitosis through different mechanisms, is able to downregulate total levels and/or phosphorylation of Bcr-Abl at the Tyr 177 site and to inactivate the Bcr-Abl downstream pathways, mediated by Lyn, CrkL or STAT3/STAT5. These effects are associated with caspase-mediated cell death. Tyr177 phosphorylation mediates Bcr-Abl downstream signaling by inducing the formation of a Lyn-Gab2-Bcr-Abl complex, and is required for Bcr-Abl-induced leukemia [26,27]. This binding results in Lyn activation by phosphorylation [28]. Lyn kinase further regulates survival and responsiveness of CML cells to inhibition of Bcr-Abl kinase [19]. Interestingly, Lyn can also phosphorylate Tyr177 in Bcr-Abl [26], resulting in a potential feedback mechanism. The Gab2-Bcr-Abl complex is mediated by Grb2 [3] and results in activation of downstream proliferative/survival pathways, such as Ras-ERK and PI3K-Akt. Gab proteins couple growth factor and cytokine receptors to downstream proteins, resulting in activation of the downstream pathways Ras-ERK, PI3K-Akt and JAK/STAT pathways [3,29]. Bcr-Abl phosphorylates and activates STAT3 and STAT5 transcription factors inducing survival and proliferation. Constitutive activation of STAT3/STAT5 is critical for the maintenance of chronic myeloid leukemia [20,30]. Bcr-Abl also binds the C-terminal Proline-rich region of the adaptor protein CrkL [30]. Bcr-Abl phosphorylates CrkL, an event needed for Bcr-Abl-induced leukemia. CrkL can enhance cell migration and Bcr-Abl-mediated leukemogenesis [6,18,31-34].

Collectively, our findings indicate that the bortezomib in combination with four different mitotic inhibitors (paclitaxel, docetaxel, vincristine and BI2536), that repress mitosis by different mechanisms (microtubule stabilization, microtubule destabilization or PLK1 inhibition) [23-25] are able to shut down Bcr-Abl activity and result in caspase-dependent cell death in TKIs-resistant (paclitaxel, BI 2536) and -sensitive (paclitaxel, docetaxel, vincristine, BI 2536) Bcr-Abl-positive cell lines. A schematic representation of these findings is presented in Figure 7 [3,6,18-20,26-34].

Our results demonstrate that regimens of bortezomib combined with mitotic inhibitors (paclitaxel, docetaxel, vincristine, BI 2536) are associated with Bcr-Abl and/or P-Bcr-Abl downregulation. Few other agents (Flavopiridol, HDAC inhibitors and the PLK1 inhibitor BI 2536) [4,23,35] have been shown to induce a significant Bcr-Abl downregulation when used in combination with imatinib. Moreover, the pan-CDK inhibitor flavopiridol [4], the heat shock protein 90 (Hsp90) antagonist 17-AAG [36] and the histone deacetylase (HDAC) inhibitor SAHA [35] were previously revealed to induce apoptosis in combination with bortezomib, an effect associated with Bcr-Abl downregulation. Although the exact mechanism of Bcr-Abl downregulation is still unclear, it seems plausible that the decrease of Bcr-Abl levels and its inactivation contribute, at least in part, to the caspase-mediated cell death induced by these combinations, including the bortezomib/mitotic inhibitors regimens.

Our results point out that a bortezomib/paclitaxel combination inhibits STAT3 and STAT5 activation. Bortezomib/BI 2536 combination similarly results in a decrease in P-STAT5 levels in K562 cells. As previously shown, Bcr-Abl phosphorylates and activates STAT3 and STAT5 transcription factors resulting in cellular survival and proliferation. Constitutive activation of STAT5 is known to be critical for the maintenance of chronic myeloid leukemia and STAT3 is also constitutively active in Bcr-Abl-positive embryonic stem cells [20,30]. Thus, cell death induced by inhibition of Bcr-Abl with imatinib in Bcr-Abl-positive cells is at least in part related to the inhibition of STAT signaling. Additionally, it is known that JAK-STAT pathway activation contributes to imatinib and nilotinib resistance in Bcr-Abl-positive progenitors [37,38]. All these findings suggest that STAT3/STAT5 signaling inhibition plays an important role in bortezomib/paclitaxel- or bortezomib/BI 2536-induced cell death, in Bcr-Abl-positive cells.

Several pathways are known to be critical downstream mediators of the Bcr-Abl pro-survival and pro-leukemogenic effects. Bcr-Abl is phosphorylated at multiple phosphorylation sites, resulting in binding/phosphorylation of downstream Bcr-Abl mediators. Phosphorylation of Tyrosine 177 induces the formation of a Lyn - Gab2 - Bcr-Abl complex, important in Bcr-Abl-induced tumorigenesis [26,27]. Lyn tyrosine kinase binding to phosphorylated and active Bcr-Abl leads to Lyn activation by phosphorylation [28]. Lyn further regulates survival and responsiveness of CML cells to inhibition of Bcr-Abl kinase [19]. Interestingly, Lyn kinase can also phosphorylate Bcr-Abl [26], resulting in a potential feedback mechanism. Additionally, Bcr-Abl phosphorylates CrkL adaptor protein, an event needed for Bcr-Abl-induced leukemia. CrkL can enhance cell migration and Bcr-Abl-mediated leukemogenesis [18,31-34]. Thus, Lyn and CrkL are key regulators and downstream mediators of Bcr-Abl-induced survival and leukemogenesis that can be inhibited by downregulation or inhibition of Bcr-Abl. Our results demonstrate that the combined treatment with bortezomib and paclitaxel is able to inhibit the activity of these important Bcr-Abl downstream mediators.

JNK activation was previously associated with apoptosis induced by bortezomib in Bcr-Abl-positive cells [39] and by bortezomib in combination with the pan-CDK inhibitor Flavopiridol in both Bcr-Abl-positive and negative leukemic cells [4]. In addition, several other studies pointed out the role of JNK activation in cell death of Bcr-Abl-positive or -negative cells [40,41]. Thus, the activation of JNK seen in our results following bortezomib/paclitaxel treatment in Bcr-Abl-positive cells may contribute to cell death.

Current inhibitors of Abl kinases, such as imatinib, dasatinib or nilotinib, have shown good results in CML treatment. However, the emergence of resistance and residual disease can eventually lead to progression of CML despite treatments [3,42]. Imatinib, dasatinib or nilotinib resistance may emerge through point mutations in Bcr-Abl (such as T315I), Bcr-Abl gene amplification and/or an increase in Bcr-Abl protein levels [4]. To investigate alternative treatments for these particular cases, we have indeed developed two different cell lines derived from K562 (K562-R) and LAMA84 (LAMA84-R) cell lines, which are completely resistant to 1μM imatinib. While the levels of Bcr-Abl and P-Bcr-Abl in LAMA84-R are much higher than in LAMA84-S cells, the levels of Bcr-Abl and P-Bcr-Abl in K562-R compared with K562-S are much closer to each other (Figure 4). Thus, the increased expression of Bcr-Abl is probably at least in part responsible for the LAMA-R resistance to imatinib, dasatinib and nilotinib, while possible mutations may be responsible for the K562-R resistance. Additionally, we have used the Baf3 Bcr-Abl T315I cell line, derived from Baf3 (an immortalized murine *pro*-B cell line), which is also resistant to 1μM imatinib and at least partially resistant to dasatinib and nilotinib treatments (Figures S4-6). In addition to its effect on imatinib-sensitive cell lines, the bortezomib/paclitaxel regimen was able to induce caspase cleavage, a measure of caspase activation [43-45], in K562-R cells and significant downregulation of the total levels and phosphorylation of Bcr-Abl in all tested TKIs-resistant cell lines. Thus, such combination may be a good strategy to treat resistant cases due to either an increase in Bcr-Abl expression or Bcr-Abl mutations that abrogate imatinib, dasatinib or nilotinib inhibitory effects. 

Notably, in addition to the bortezomib/paclitaxel regimen, our results demonstrate that bortezomib, in combination with other mitotic inhibitors that act by inducing mitotic arrest through various mechanisms (microtubule stabilization: docetaxel, microtubule destabilization: vincristine and PLK1 inhibition: BI 2536), inhibits Bcr-Abl and results in caspase 3 activation. It has previously been established that inhibition of Bcr-Abl (Imatinib [23]) or knock-down of Bcr-Abl (shRNA for Bcr-Abl [46]) induces caspase activation and apoptosis. Thus, our results indicate that Bcr-Abl down-modulation contributes, at least in part, to caspase activation and induction of cell death. Both docetaxel and vincristine are FDA-approved for the treatment of several malignancies, alone or in combination [47,48]. Interestingly, a recent study concluded that BI 2536 has growth inhibitory effects on Bcr-Abl-positive cells that are not amplified by bortezomib after 16h of co-treatment [23]. In contrast, we are showing here that the combined treatment of bortezomib 9nM with BI 2536 8nM for 60h is significantly more effective in inducing caspase activation, PARP cleavage and cell death compared with single treatments, in both K562 and K562-R cells (Figure 5). The longer time needed for bortezomib to amplify the effects of BI 2536 might be explained by the involvement of transcriptional mechanisms in bortezomib/BI 2536-induced cell death, although further experiments are needed to clarify this aspect.

Recently, two other drugs were approved by FDA for the treatment of patients with CML whose tumors are resistant to or who cannot tolerate Imatinib, Dasatinib or Nilotinib therapies: bosulif (bosutinib) and synribo (omacetaxine mepesuccinate) [49]. Bosutinib, approved on September 4, 2012, is a TKI inhibitor efficient against many Bcr-Abl mutations, except T315I [49,50]. Omacetaxine mepesuccinate, approved on October 26, 2012, is a non-TKI drug intended to be used when leukemia progresses after therapy with at least two TKIs [49,51]. While the drug can be used for the treatment of CML patients with T315I mutation, it shows significant hematologic toxicity (grade 3-4) in clinical trials: thrombocytopenia (76% of cases), neutropenia (44% of cases), and anemia (39% of cases) [49,51]. While these two new approved drugs offer an option for many patients with imatinib, dasatinib and nilotinib-resistant CML, novel better strategies have to be developed. In contrast with bosutinib, our combined treatment with bortezomib and mitotic inhibitors is able to target Bcr-Abl with T315I mutation. Moreover, lower concentrations of each drug can be used in synergistic combinations, which may reduce toxicity. However, the toxicity of our regimens remains to be established.

The potential of Bortezomib or Bortezomib-based combination therapies in hematological malignancies is also underscored by their ability to target tumor environment [52,53]. Tumor microenvironment is a dominant force in inducing resistance to therapy in multiple malignancies [54]. Tumor microenvironment plays a key role in leukemic stem cell maintenance [55] and in modulating signal transduction and resistance in CML [55] and AML [56].

In conclusion, the combination of bortezomib and mitotic inhibitors such as paclitaxel, docetaxel, vincristine or BI 2536 is an effective strategy for targeting of both TKIs -resistant and -sensitive Bcr-Abl-positive leukemic cells. These regimens are able to inhibit Bcr-Abl activity and its downstream signaling, and to activate caspase-dependent cell death. In addition, these regimens are able to overcome the resistance to imatinib, dasatinib and nilotinib, brought about by Bcr-Abl protein overexpression or Bcr-Abl mutations (including T315I), making them attractive potential therapies for Bcr-Abl-positive leukemias such as CML, especially for those resistant to current treatments. As the combined treatment is also efficient in non-CML Bcr-Abl positive cells such as the Baf3 Bcr-Abl cell line, it may also be a promising therapy for non-CML Ph+ leukemias (Ph+ALL, Ph+AML).

## Supporting Information

Figure S1 **Combined treatment of bortezomib and paclitaxel induces cell death in human leukemic Bcr-Abl-positive K562 cell line (in support of Figure 1a and b).** **A**. K562 leukemic cells were exposed to bortezomib (9nM) with or without paclitaxel (6nM) for 48h. The percentage of cell death was measured using PE-Annexin V/7-AAD staining and flow cytometry as described in “Materials and Methods”. A representative experiment from three individual experiments is shown here. **B**. K562 leukemic cells were exposed to bortezomib (10nM), paclitaxel (5nM) or the combination for 48h. Viability was measured using the MTT assay as described in “Materials and Methods”. The results represent the mean +/- standard deviations (SDs) of a representative experiment. “***” = p<0.0001. **C**. K562 cells were treated with bortezomib (9nM), paclitaxel (6nM) or the combination, for 40h. The percentage of proliferating cells was measured by BrdU incorporation assay (cells were exposed to BrdU for 90 min before fixation and permeabilization), as described in “Materials and Methods” section. A representative experiment from three individual experiments is shown here.
(TIF)

Figure S2 **Bortezomib and paclitaxel synergistically induce cell death in K562 (a) and LAMA84 (b) Bcr-Abl leukemic cells (in support of Figure 1).** **A**. K562 cells were treated with increasing concentrations of each drug alone and in combination, maintaining the same concentration ratio of bortezomib : paclitaxel 1.6 : 1. Calculated Combination Index (CI) using the Chou-Talalay method is below 1 when the affected fraction fa>0.1=10%, which demonstrates the synergism of the combined bortezomib/paclitaxel treatment. A representation of the calculated CI for a range of affected fractions from 0.1 to 0.8 is shown. **B**. LAMA84 cells were treated with increasing concentrations of each drug alone and in combination, maintaining the same concentration ratio of bortezomib: paclitaxel 1: 1.5. The calculated Combination Index (CI) using the Chou-Talalay method is below 1, which demonstrates the synergism of the combined bortezomib/paclitaxel treatment. A representation of the calculated CI for a range of affected fractions from 0.1 to 1 is shown.
(TIF)

Figure S3 **Combined treatment with 8nM bortezomib and 5 nM paclitaxel induces activation of p38, but not of EKR (in support of Figure 2).** K562 leukemic cells were treated with 9nM bortezomib and 6nM paclitaxel for 48h, followed by detection of the total and phosphorylated protein levels of p38MAPK and ERK 1&2. The combined regimen does induces a change in phosphorylation of the P-ERK 1&2 (A), but results in a strong increase in p38 phosphorylation (B). β-Actin was used as a loading control.
(TIF)

Figure S4 **K562-R cells are resistant to imatinib, nilotinib and dasatinib treatments (in support of Figures 4a and 5).** K562 (K562-S) and imatinib-resistant K562-R cells were plated in 25cm^2^ flasks (0.6-0.8 x 10^6^ cells/10 ml/flask) and treated with 0.5 µM imatinib (Imat), 0.9 µM imatinib, 0.125 µM nilotinib (Nilot) or 0.0025 µM dasatinib (Dasat) for 48h. Viability was measured by Trypan Blue dye exclusion method, using a TC10 Automated Cell Counter (Biorad, USA). Results represent the mean +/- SDs of 6 measurements/condition for the representative experiment presented in Figure 4a. A total of three independent experiments were performed; “***” = p<0.0001; .
(TIF)

Figure S5 **LAMA84-R cells are resistant to imatinib, nilotinib, dasatinib treatments (in support of Figure 4b).** LAMA84 (LAMA84-S) and imatinib-resistant LAMA84-R cells were plated in 25cm^2^ flasks (0.7 x 10^6^ cells/10 ml/flask) and treated with 0.9 µM imatinib, 0.125 µM nilotinib or 0.005 µM dasatinib for 48h. Viability was measured by Trypan Blue dye exclusion method, using a TC10 Automated Cell Counter (Biorad, USA). Results represent the mean +/- SDs of 8 measurements/condition for the representative experiment presented in Figure 4b. A total of three independent experiments were performed; “***” = p<0.0001;.
(TIF)

Figure S6 **Baf3 Bcr-Abl T315 cells are resistant to imatinib, nilotinib, dasatinib treatments (in support of Figure 4c).** Murine Baf3 Bcr-Abl and Baf3 Bcr-Abl T315I cells were plated in 75cm^2^ flasks (4 x 10^6^ cells/35 ml/flask) and treated with 0.5 or 1 µM imatinib. For nilotinib and dasatinib treatments, the cells were plated in 25cm^2^ flasks (2 x 10^6^ cells/10 ml/flask) and treated with 0.125 µM or 0.5 µM nilotinib and 0.056 µM or 0.112 µM dasatinib for 48h. Viability was measured by Trypan Blue dye exclusion method, using a TC10 Automated Cell Counter (Biorad, USA). Results represent the mean +/- SDs of 6 measurements/condition for the representative experiment presented in Figure 4c. A total of three independent experiments were performed; “***” = p<0.0001; .
(TIF)

Figure S7 **Bortezomib and paclitaxel synergistically induce cell death in K562-R cells.** K562-R cells were treated with increasing concentrations of each drug alone and in combination, maintaining the same concentration ratio of bortezomib : paclitaxel 1.5 : 1. Calculated Combination Index (CI) using the Chou-Talalay method is below 1 when the affected fraction fa>0.2=10%, which demonstrates the synergism of the combined bortezomib/paclitaxel treatment. A representation of the calculated CI for a range of affected fractions from 0.1 to 0.9 is shown.
(TIF)
