# Supplementary figures and images for: Combination of Bortezomib and Mitotic Inhibitors Down-Modulate Bcr-Abl and Efficiently Eliminates Tyrosine-Kinase Inhibitor Sensitive and Resistant Bcr-Abl-Positive Leukemic Cells
Source: PLoS One. 2013 Oct 14;8(10):e77390. doi: 10.1371/journal.pone.0077390 (PMC3796452; doi:10.1371/journal.pone.0077390)

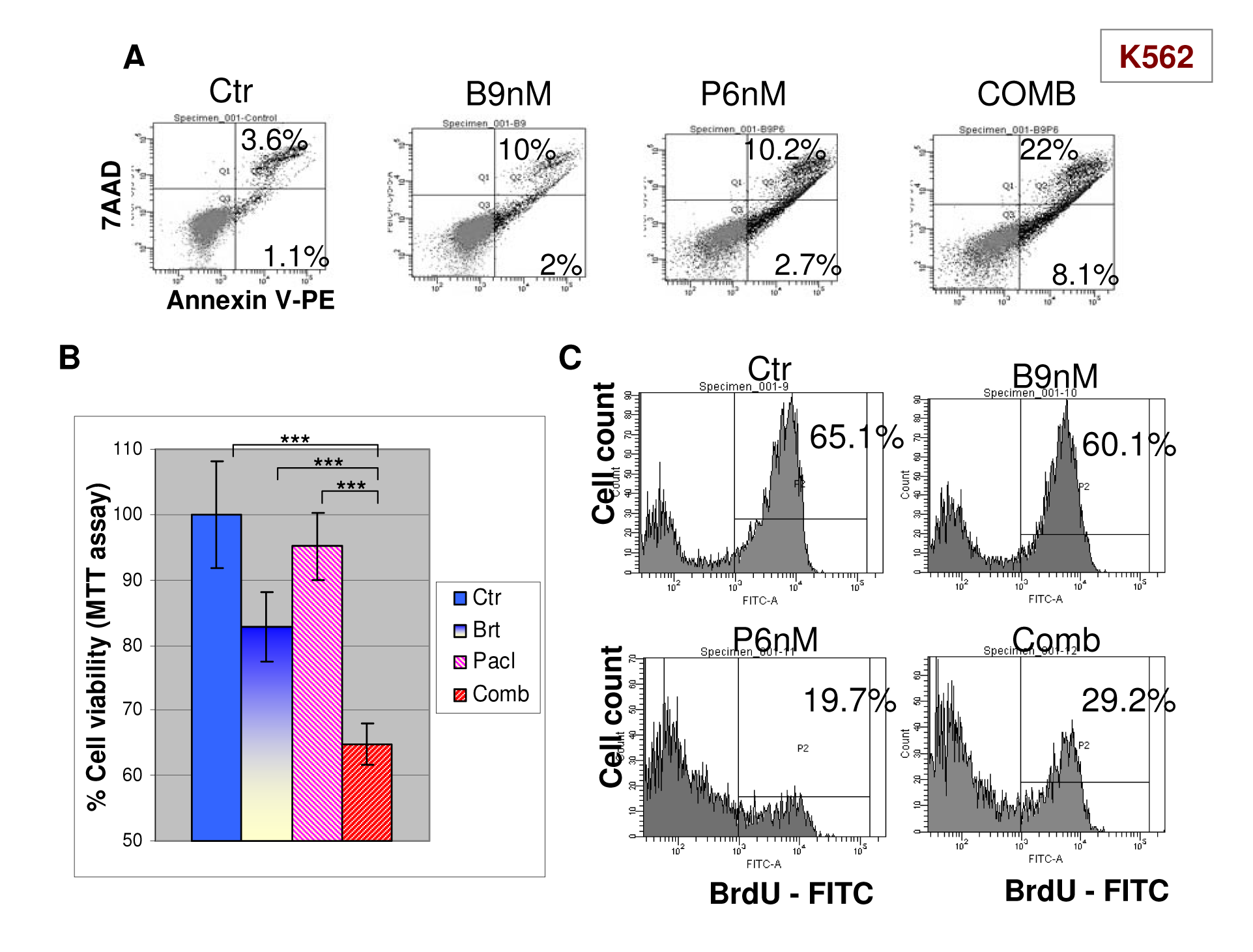

Supplement: Figure S1 — Combined treatment of bortezomib and paclitaxel induces cell death in human leukemic Bcr-Abl-positive K562 cell line (in support of Figure 1a and b). A. K562 leukemic cells were exposed to bortezomib (9nM) with or without paclitaxel (6nM) for 48h. The percentage of cell death was measured using PE-Annexin V/7-AAD staining and flow cytometry as described in “Materials and Methods”. A representative experiment from three individual experiments is shown here. B. K562 leukemic cells were exposed to bortezomib (10nM), paclitaxel (5nM) or the combination for 48h. Viability was measured using the MTT assay as described in “Materials and Methods”. The results represent the mean +/- standard deviations (SDs) of a representative experiment. “***” = p<0.0001. C. K562 cells were treated with bortezomib (9nM), paclitaxel (6nM) or the combination, for 40h. The percentage of proliferating cells was measured by BrdU incorporation assay (cells were exposed to BrdU for 90 min before fixation and permeabilization), as described in “Materials and Methods” section. A representative experiment from three individual experiments is shown here. (TIF) [file pone.0077390.s001.tif]

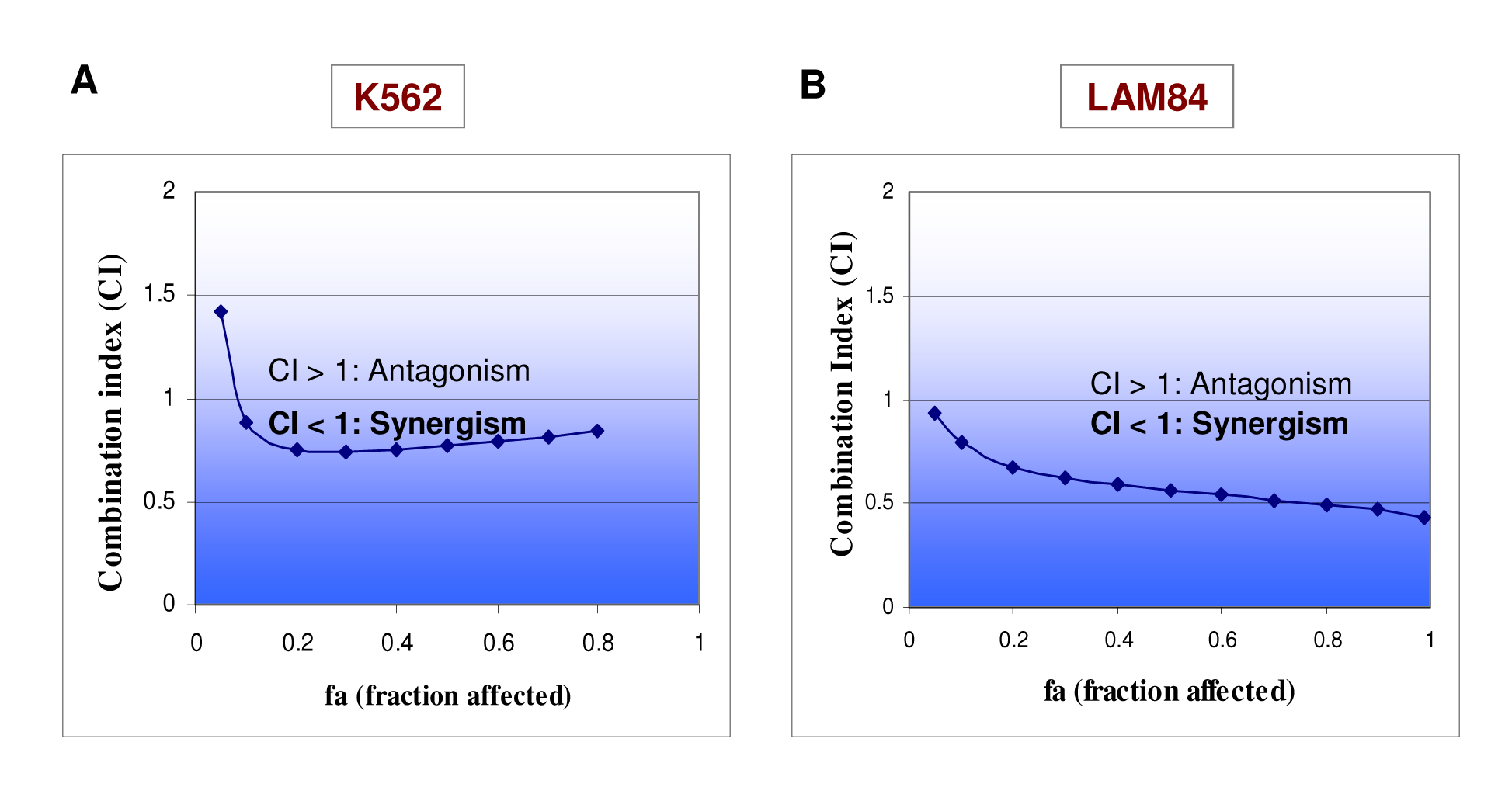

Supplement: Figure S2 — Bortezomib and paclitaxel synergistically induce cell death in K562 (a) and LAMA84 (b) Bcr-Abl leukemic cells (in support of Figure 1). A. K562 cells were treated with increasing concentrations of each drug alone and in combination, maintaining the same concentration ratio of bortezomib : paclitaxel 1.6 : 1. Calculated Combination Index (CI) using the Chou-Talalay method is below 1 when the affected fraction fa>0.1=10%, which demonstrates the synergism of the combined bortezomib/paclitaxel treatment. A representation of the calculated CI for a range of affected fractions from 0.1 to 0.8 is shown. B. LAMA84 cells were treated with increasing concentrations of each drug alone and in combination, maintaining the same concentration ratio of bortezomib: paclitaxel 1: 1.5. The calculated Combination Index (CI) using the Chou-Talalay method is below 1, which demonstrates the synergism of the combined bortezomib/paclitaxel treatment. A representation of the calculated CI for a range of affected fractions from 0.1 to 1 is shown. (TIF) [file pone.0077390.s002.tif]

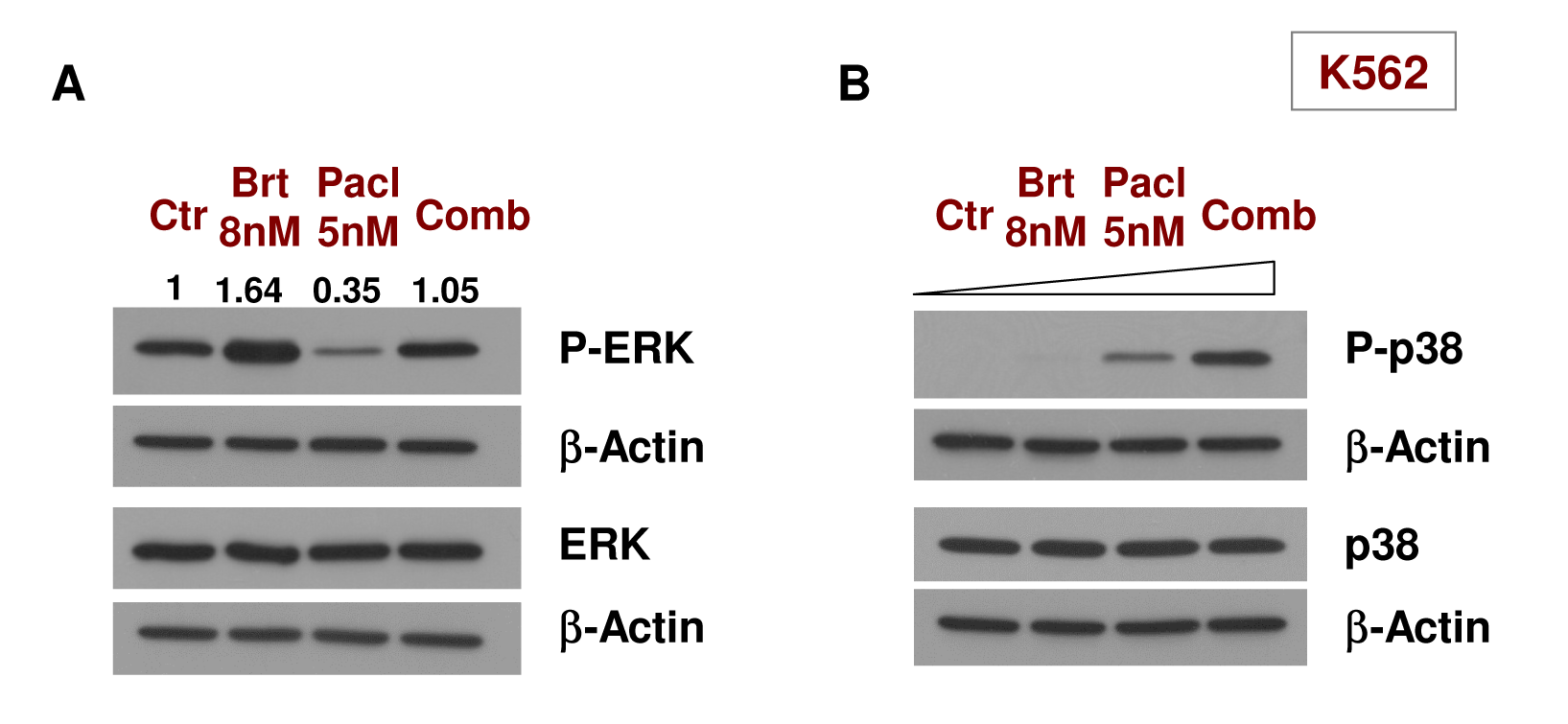

Supplement: Figure S3 — Combined treatment with 8nM bortezomib and 5 nM paclitaxel induces activation of p38, but not of EKR (in support of Figure 2). K562 leukemic cells were treated with 9nM bortezomib and 6nM paclitaxel for 48h, followed by detection of the total and phosphorylated protein levels of p38MAPK and ERK 1&2. The combined regimen does induces a change in phosphorylation of the P-ERK 1&2 (A), but results in a strong increase in p38 phosphorylation (B). β-Actin was used as a loading control. (TIF) [file pone.0077390.s003.tif]

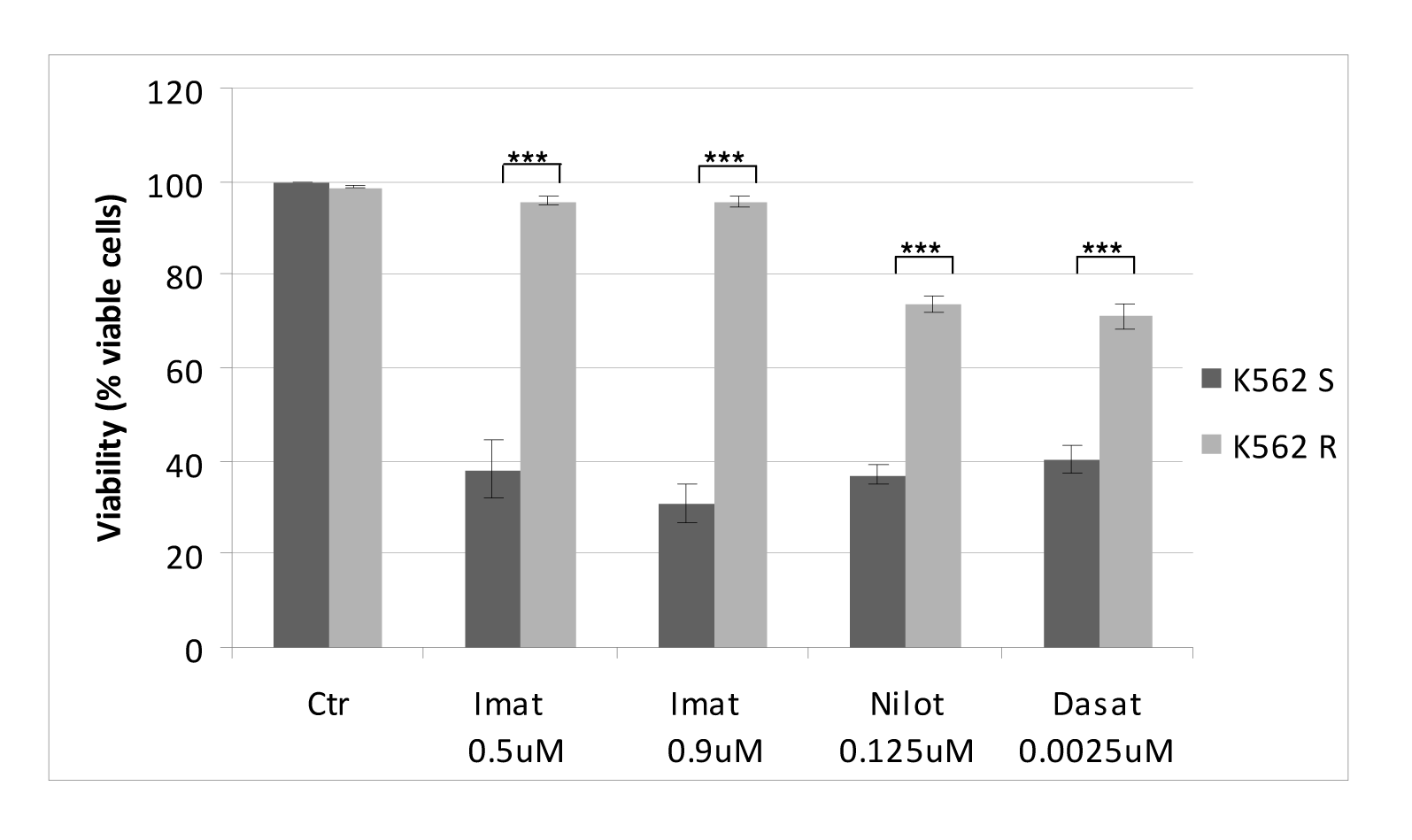

Supplement: Figure S4 — K562-R cells are resistant to imatinib, nilotinib and dasatinib treatments (in support of Figures 4a and 5). K562 (K562-S) and imatinib-resistant K562-R cells were plated in 25cm2 flasks (0.6-0.8 x 10^6^ cells/10 ml/flask) and treated with 0.5 µM imatinib (Imat), 0.9 µM imatinib, 0.125 µM nilotinib (Nilot) or 0.0025 µM dasatinib (Dasat) for 48h. Viability was measured by Trypan Blue dye exclusion method, using a TC10 Automated Cell Counter (Biorad, USA). Results represent the mean +/- SDs of 6 measurements/condition for the representative experiment presented in Figure 4a. A total of three independent experiments were performed; “***” = p<0.0001; . (TIF) [file pone.0077390.s004.tif]

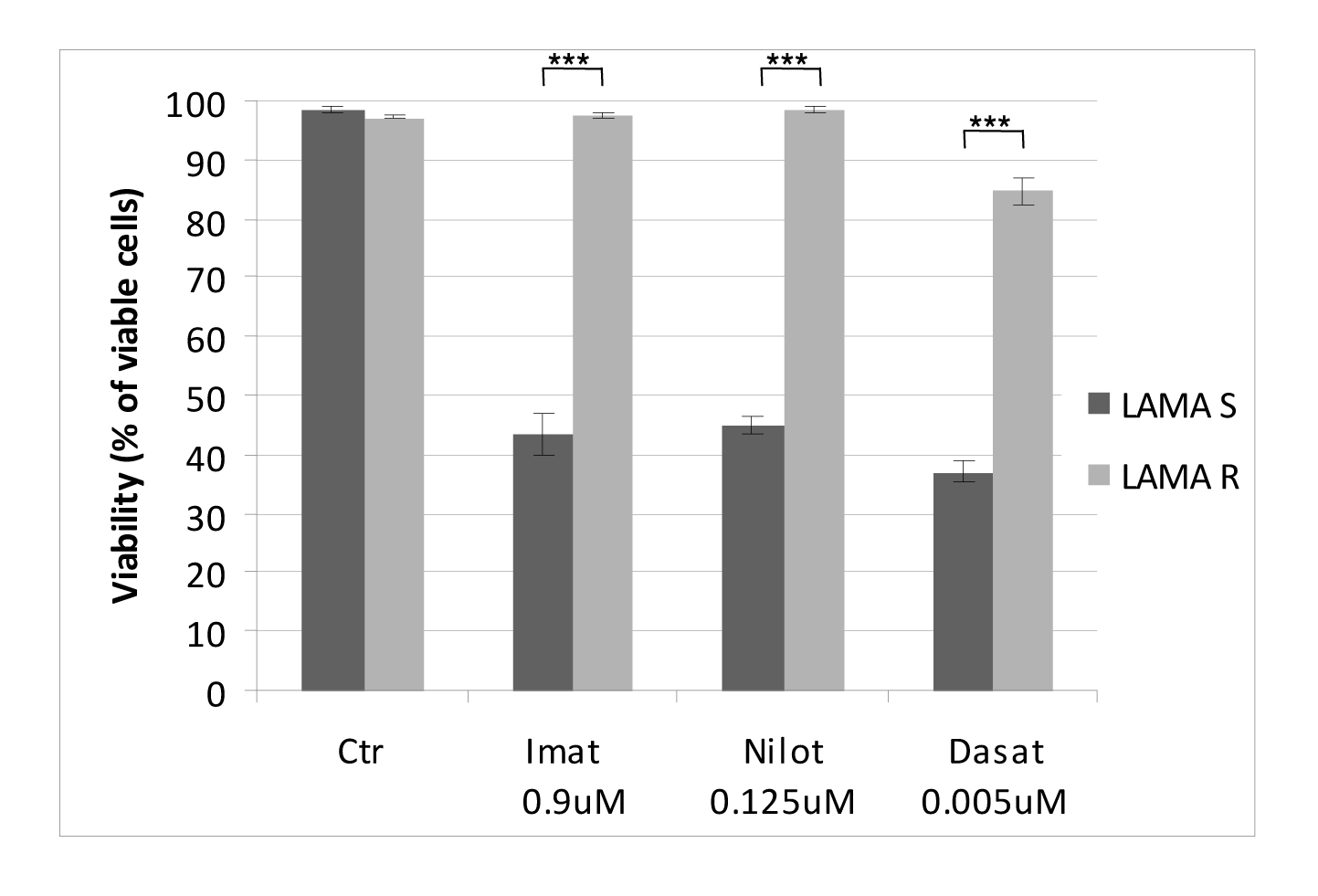

Supplement: Figure S5 — LAMA84-R cells are resistant to imatinib, nilotinib, dasatinib treatments (in support of Figure 4b). LAMA84 (LAMA84-S) and imatinib-resistant LAMA84-R cells were plated in 25cm2 flasks (0.7 x 10^6^ cells/10 ml/flask) and treated with 0.9 µM imatinib, 0.125 µM nilotinib or 0.005 µM dasatinib for 48h. Viability was measured by Trypan Blue dye exclusion method, using a TC10 Automated Cell Counter (Biorad, USA). Results represent the mean +/- SDs of 8 measurements/condition for the representative experiment presented in Figure 4b. A total of three independent experiments were performed; “***” = p<0.0001;. (TIF) [file pone.0077390.s005.tif]

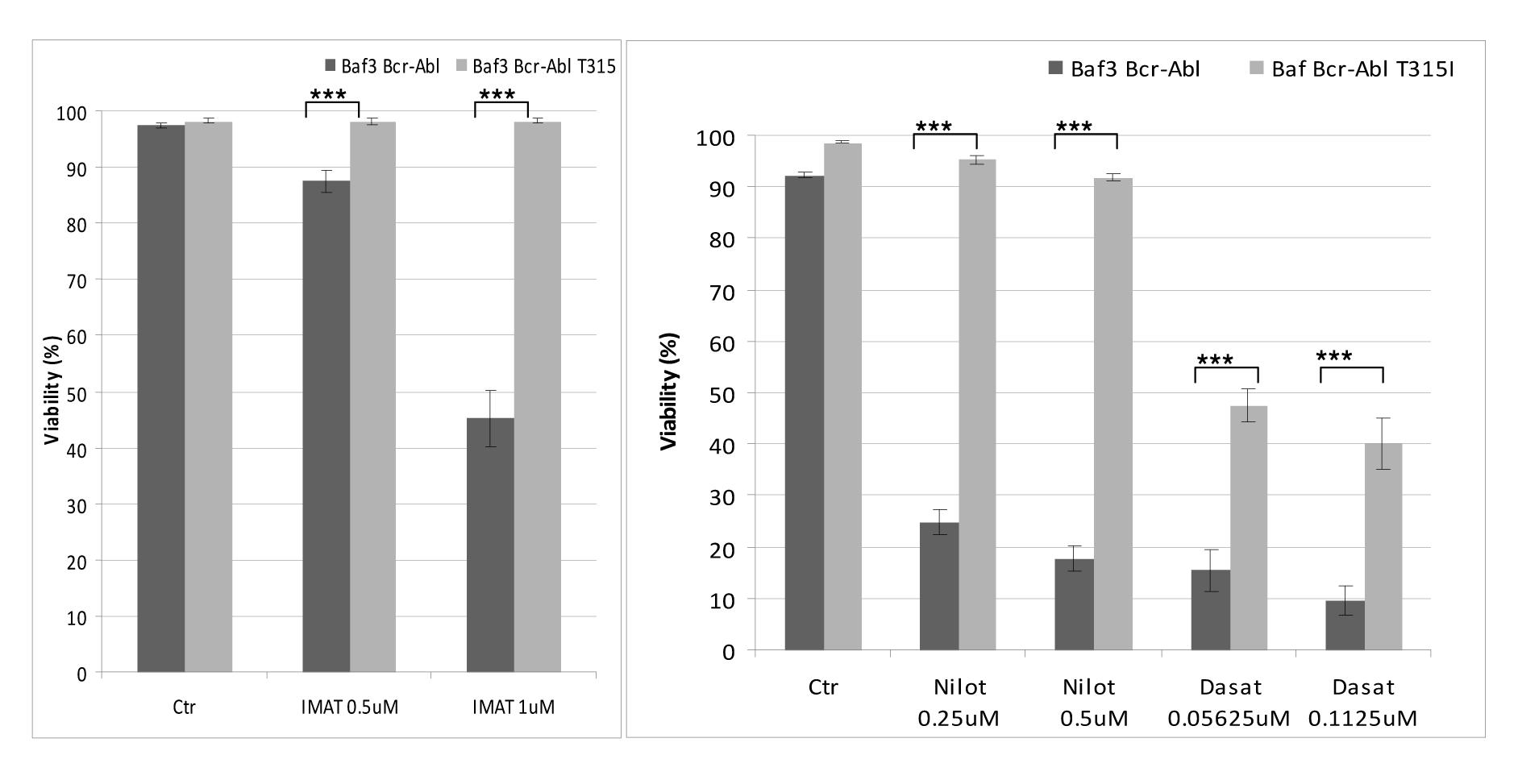

Supplement: Figure S6 — Baf3 Bcr-Abl T315 cells are resistant to imatinib, nilotinib, dasatinib treatments (in support of Figure 4c). Murine Baf3 Bcr-Abl and Baf3 Bcr-Abl T315I cells were plated in 75cm2 flasks (4 x 10^6^ cells/35 ml/flask) and treated with 0.5 or 1 µM imatinib. For nilotinib and dasatinib treatments, the cells were plated in 25cm2 flasks (2 x 10^6^ cells/10 ml/flask) and treated with 0.125 µM or 0.5 µM nilotinib and 0.056 µM or 0.112 µM dasatinib for 48h. Viability was measured by Trypan Blue dye exclusion method, using a TC10 Automated Cell Counter (Biorad, USA). Results represent the mean +/- SDs of 6 measurements/condition for the representative experiment presented in Figure 4c. A total of three independent experiments were performed; “***” = p<0.0001; . (TIF) [file pone.0077390.s006.tif]

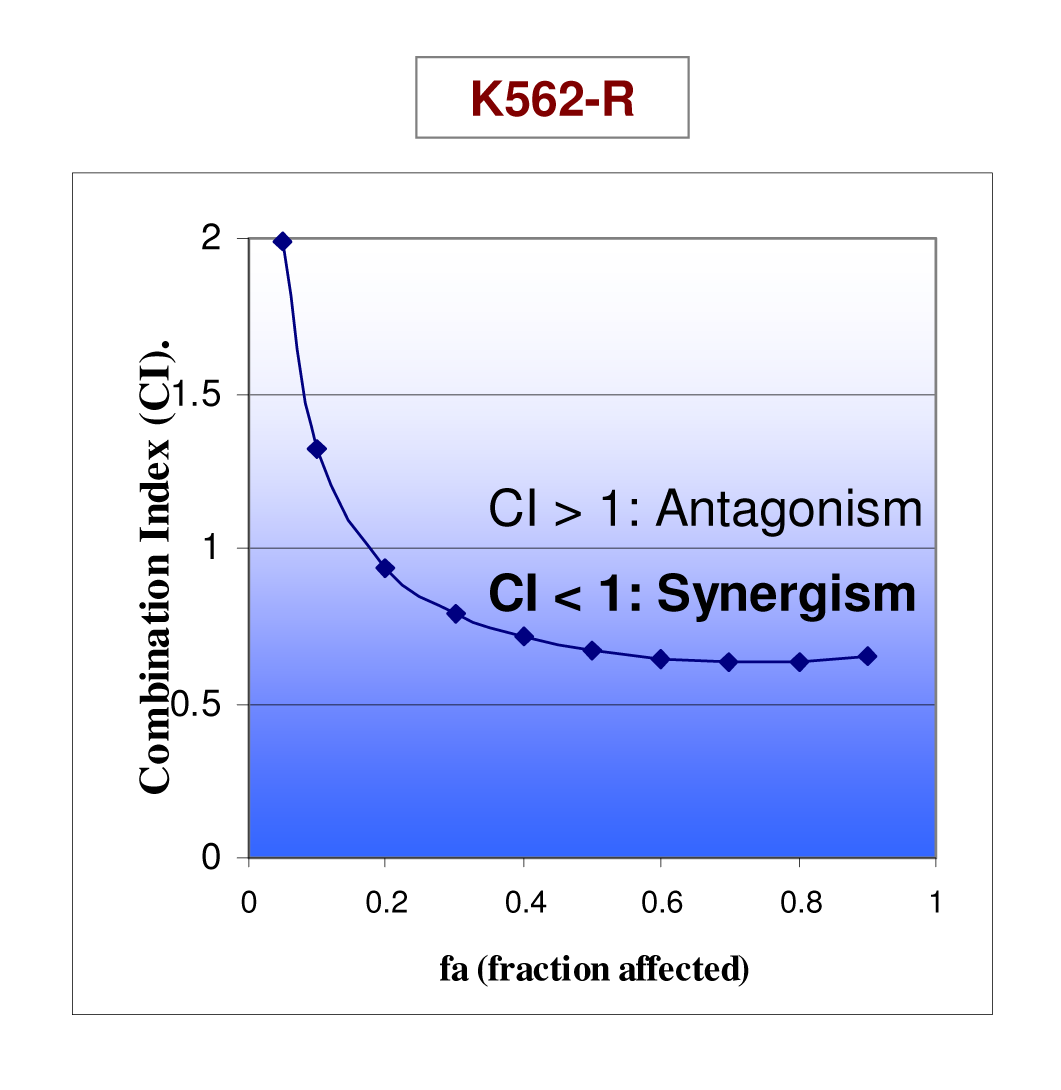

Supplement: Figure S7 — Bortezomib and paclitaxel synergistically induce cell death in K562-R cells. K562-R cells were treated with increasing concentrations of each drug alone and in combination, maintaining the same concentration ratio of bortezomib : paclitaxel 1.5 : 1. Calculated Combination Index (CI) using the Chou-Talalay method is below 1 when the affected fraction fa>0.2=10%, which demonstrates the synergism of the combined bortezomib/paclitaxel treatment. A representation of the calculated CI for a range of affected fractions from 0.1 to 0.9 is shown. (TIF) [file pone.0077390.s007.tif]
